# Nuclear AhR and membranous PD-L1 in predicting response of non-small cell lung cancer to PD-1 blockade

**DOI:** 10.1038/s41392-023-01416-5

**Published:** 2023-05-29

**Authors:** Si-Chong Han, Gui-Zhen Wang, Ya-Ning Yang, Wen-Feng Fang, Bei-Bei Sun, Jian-Dong Zhang, Hua-Qiang Zhou, Li Zhang, Yan Wang, Guang-Biao Zhou

**Affiliations:** 1 State Key Laboratory of Molecular Oncology and Department of Internal Medicine, National Cancer Center/National Clinical Research Center for Cancer/Cancer Hospital, Chinese Academy of Medical Sciences and Peking Union Medical College, Beijing, China; 2 Lung Cancer Research Centre and State Key Laboratory of Oncology in South China, Sun Yat-Sen University Cancer Center, Guangzhou, China; 3 Shanxi Bethune Hospital Affiliated with Shanxi Academy of Medical Sciences, Taiyuan, China

**Keywords:** Lung cancer, Prognostic markers

**Dear Editor**,

Programmed cell death 1 ligand 1 (PD-L1) has been used as a biomarker for immune checkpoint inhibitors (ICIs) which exert durable efficacy in non-small cell lung cancer (NSCLC).^1,2^ However, many PD-L1-high patients only marginally respond to, and PD-L1-low patients still benefit from, ICIs.^3,4^ Transcription factor aryl hydrocarbon receptor (AhR) plays critical roles in development and function of both innate and adaptive immune cells,^5,6^ controls the expression of PD-L1 in lung epithelial cells and is associated with patient response to ICIs.^7^ Here, we tested whether or not AhR and AhR nuclear translocator (ARNT) could enhance PD-L1 prognosis predicting value in NSCLCs upon ICI treatment.

Between July 2016 and November 2018, we retrospectively acquired 65 pre-ICI treatment formalin-fixed, paraffin-embedded (FFPE) specimens from NSCLC patients (Supplementary Table 1) who were then treated with pembrolizumab. In this training cohort, the proportion of cells with multiple positive staining, i.e., nucleus-localized AhR (AhR^N^), cytoplasmic AhR (AhR^C^), nucleus-localized ARNT (ARNT^N^), membrane-localized PD-L1 (PD-L1^M^) and nucleus-localized PD-L1^N^ (Fig. 1a), was evaluated by multiplex immunohistochemistry (mIHC). The optimum density cutoff values of PD-L1^M^, AhR^N^, AhR^N^ARNT^N^, AhR^N^PD-L1^M^ (Supplementary Fig. 1a–d), AhR^C^, PD-L1^N^, ARNT^N^, AhR^N^PD-L1^N^, AhR^C^PD-L1^M^ (Supplementary Fig. 2a–e), AhR^C^PD-L1^N^, AhR^C^ARNT^N^, ARNT^N^PD-L1^M^, and ARNT^N^PD-L1^N^ (Supplementary Fig. 3a–d) were determined based on progression-free survival (PFS) of the patients. A high proportion of PD-L1^M+^ cells indicated a better response to immunotherapy (Fig. 1b; HR: 0.226, 95% CI: 0.0759-0.675), consistent with previous studies.^1^ Notably, the densities of AhR^N^ (HR: 0.139, 95% CI: 0.0319–0.605), AhR^N^ARNT^N^ (HR: 0.109, 95% CI: 0.0218–0.547) and AhR^N^PD-L1^M^ (HR: 0.0831, 95% CI: 0.0166–0.417) (Supplementary Fig. 1b; Fig. 1b) effectively predicted patient PFS. Considering the ability to effectively distinguish responders, PD-L1^M^, AhR^N^PD-L1^M^, AhR^N^ARNT^N^, AhR^N^, PD-L1^N^, ARNT^N^PD-L1^N^ and AhR^C^PD-L1^N^ were selected for further model construction. Due to the strong multicollinearity among the 7 variables (Supplementary Fig. 4a), we implemented LASSO logistic regression with λ = 0.04 (Supplementary Fig. 4b) to determine the variables most relevant to PFS. If the density of one variable exceeded its cutoff, the status of this variable was equivalent to 1; otherwise, the status was equivalent to 0. Five robust immunotherapeutic markers were identified (Supplementary Fig. 4c), and the formula that reflects the risk of NSCLC progression was risk score = -(1.919 × AhR^N^PD-L1^M^ status)—(0.119 × AhR^N^ARNT^N^ status)—(0.696 × PD-L1^M^ status) + (0.689 × ARNT^N^PD-L1^N^ status) + (0.343 × AhR^C^PD-L1^N^ status). The risk score plots suggested that AhR^N^PD-L1^M^, AhR^N^ARNT^N^ and PD-L1^M^ were favorable prognostic factors, while ARNT^N^PD-L1^N^ and AhR^C^PD-L1^N^ were negative prognostic factors (Supplementary Fig. 4d).

**Fig. 1:**
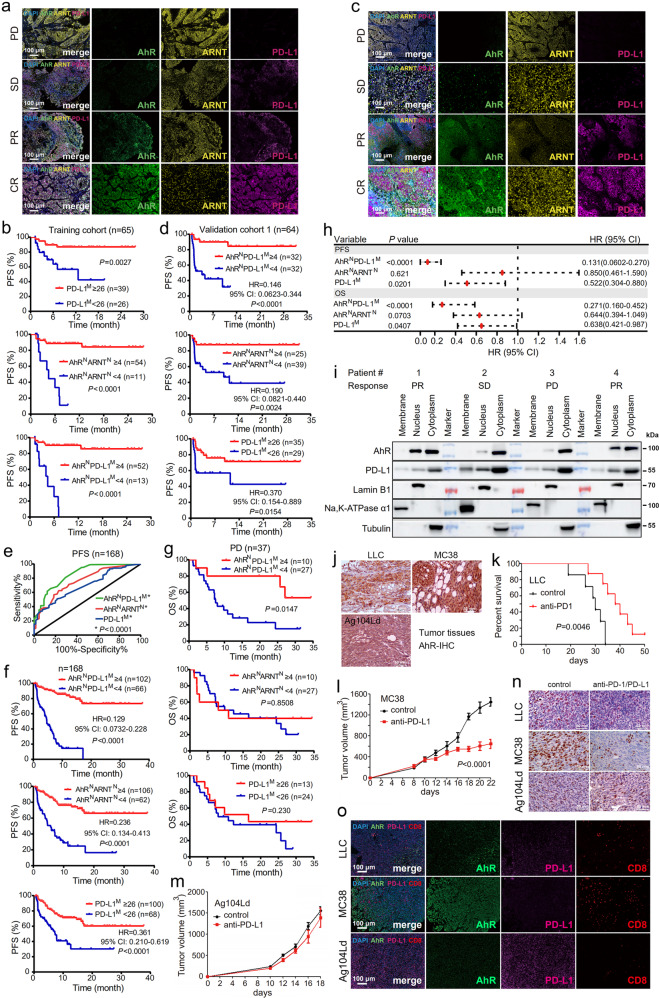
AhR^N^PD-L1^M^ is more precise than PD-L1^M^ in predicting the prognosis of NSCLC patients treated with immune checkpoint inhibitors. **a** Representative mIHC images of NSCLC patients with differential responses to immunotherapy in the training cohort. PD, progression of disease. SD, stable disease. PR, partial response. CR, complete response. Scale bars = 100 μm. **b** The Kaplan-Meier survival curve of patient PFS corresponding the optimum density cutoff of PD-L1^M^, AhR^N^ARNT^N^ and AhR^N^PD-L1^M^. ^M^, membrane-localized; ^N^, nucleus-localized. **c** Representative mIHC images of NSCLC patients with differential responses to immunotherapy in the validation cohort 1. **d** The Kaplan-Meier survival curve of patient PFS in the validation cohort 1. **e** ROC curve based on PFS of all the 168 patients according to the status of AhR^N^PD-L1^M^, AhR^N^ARNT^N^, and PD-L1^M^. **f** The Kaplan-Meier survival curve of PFS of all the 168 patients. **g** The Kaplan-Meier survival curve of OS of 37 patients who developed progression of disease to PD-1 blocker. **h** Multivariable Cox regression analyses of the significant variables selected by univariate Cox regression analyses of AhR^N^PD-L1^M^, AhR^N^ARNT^N^, PD-L1^M^, clinicopathological characteristics, and patient survival. The error bars indicate 95% confidence interval (CI). **i** The expression level of AhR and PD-L1 in nuclear, cytoplasmic, and membranous compartments of tumor samples of NSCLC patients receiving Keytruda treatment, tested by western blot. **j** Immunohistochemistry (IHC) assays of AhR expression in tumor samples harvested from mice inoculated with indicated murine cancer cells. Scale bar = 50 μm. **k** The survival curve of LLC cells-bearing C57BL/6 mice, estimated by the Kaplan-Meier analysis and Log-rank test. **l** Tumor volume of MC38 cells-bearing C57BL/6 mice that were treated with or without anti-PD-L1 antibody. **m** Tumor volume of Ag104Ld cells-bearing B6C3F1 mice that were treated with or without anti-PD-L1 antibody. **n** IHC analysis of Ki67 in tumor samples of mice that were inoculated with indicated murine cancer cells and treated with or without anti-PD-1/anti-PD-L1 antibody. Scale bar = 50 μm. **o** Representative mIHC images of tumor tissues from mice that were inoculated with indicated murine cancer cells. The statistical significance was assessed by two-sided Student’s *t* test and *P* values of Kaplan-Meier survival analysis were calculated by log-rank test

After confirming that AhR^N^PD-L1^M^-, AhR^N^ARNT^N^- and PD-L1^M^-positive cells were concentrated in responders (Supplementary Fig. 5a), the 3 factors were selected for further comparison. The distribution of all clinicopathological characteristics except smoking status showed no significant differences between the high- and low-proportion groups (Supplementary Table 2 and Supplementary Fig. 5b, c). Similarly, the OS probabilities of NSCLC patients receiving immunotherapy showed salient discrepancies between groups divided according to the cutoff values of AhR^N^PD-L1^M^ (HR: 0.160, 95% CI: 0.0375–0.680), AhR^N^ARNT^N^ (HR: 0.182, 95% CI: 0.0418–0.794) and PD-L1^M^ (HR: 0.401, 95% CI: 0.176–0.909) (Supplementary Fig. 6a). ROC analysis was carried out and the area under the receiver operating characteristic curve (AUC) indicated that AhR^N^PD-L1^M^, AhR^N^ARNT^N^ and PD-L1^M^ could validly predict patient PFS (Supplementary Fig. 6b), and Delong test showed that AhR^N^PD-L1^M^ was the most powerful one (*Z* = 2.444, *P* = 0.0145).

To verify our findings in training group, two validation cohorts were employed (Supplementary Tables 1, 3, 4). Similar to that of training cohort, the distribution of AhR^N^PD-L1^M^-, AhR^N^ARNT^N^- and PD-L1^M^-positive cells (Fig. 1c) was related to smoking status (Supplementary Table 3 and 4, Supplementary Fig. 7a, b and Supplementary Fig. 8a, b). The factors divided patients into two group with significantly different PFS and OS, and AhR^N^PD-L1^M^ was the most accurate one (Fig. 1d, Supplementary Fig. 7c and Supplementary Fig. 8c, d).

The three cohorts were pooled to test the predictive ability of these factors and the results showed that compared to AhR^N^ARNT^N^ (AUC: 0.764; 95% CI: 0.692–0.837) and PD-L1^M^ (AUC: 0.702; 95% CI: 0.624-0.78), AhR^N^PD-L1^M^ had the largest AUC (0.857; 95% CI: 0.799–0.915; Fig. 1e), and Delong test confirmed AhR^N^PD-L1^M^ as the most accurate predictor (*Z* = 3.116, *P* = 0.00183). AhR^N^PD-L1^M^ possessed the highest accuracy in predicting PFS (Fig. 1f) and OS (Supplementary Fig. 9), in short-term and long-term observations (Supplementary Fig. 10), and in the vast majority of cases when stratified by clinicopathological factors (Supplementary Fig. 11a). Net reclassification index (NRI) for PFS was calculated and the result was 0.297 (*Z* = 3.094, *P* = 0.00197), suggesting that the proportion of exact classification increased from PD-L1^M^ to AhR^N^PD-L1^M^ by 29.7%. NRI for OS was 0.245 (Z = 2.880, *P* = 0.00398), indicating that the proportion of exact classification increased by 24.5%. Among 37 patients who developed PD upon immunotherapy, higher level of AhR^N^PD-L1^M^ was associated with longer OS (Fig. 1g). Univariate analysis showed that AhR^N^PD-L1^M^, AhR^N^ARNT^N^ and PD-L1^M^ were associated with patient PFS and OS (Supplementary Fig. 11b). Multivariable Cox regression analysis showed that AhR^N^PD-L1^M^ and PD-L1^M^ were independent prognostic indicators for NSCLCs upon PD-1 blockade, and AhR^N^PD-L1^M^ was more powerful (Fig. 1h).

To verify the findings by mIHC, we tested the expression of AhR and PD-L1 by western blot. In 15 patients who had enough frozen tumor samples, AhR was detected in nuclear and cytoplasmic compartments, while PD-L1 was detected in membrane, cytoplasm, and nucleus (Fig. 1i, Supplementary Fig. 12a), and the higher expression levels of AhR^N^, the more likely the patients would benefit from immunotherapy (*P* = 0.012). However, PD-L1^M^ could not distinguish good response from poor response in these patients (Supplementary Fig. 12b).

We established xenograft murine models to validate the findings in patient samples, and found that tumor tissues of mice injected with murine lung cancer LLC and colon cancer MC38 cells exhibited high level AhR^N^, whereas murine fibrosarcoma Ag104Ld cells-injected mice had low level AhR^N^ (Fig. 1j). The anti-PD-1/anti-PD-L1 antibody exerted an obvious antitumor effect on mice injected with LLC (Fig. 1k, Supplementary Fig. 13a) and MC38 cells (Fig. 1l, Supplementary Fig. 13b), but not on Ag104Ld-bearing mice (Fig. 1m, Supplementary Fig. 13c, d). The effects of PD-1/PD-L1 inhibition were confirmed by IHC analysis of cell proliferation marker Ki67 (Fig. 1n). mIHC staining of tumor specimens showed that LLC and MC38 cells expressed high level AhR^N^ and moderate level PD-L1^M^, while Ag104Ld cells expressed high level PD-L1^M^ and low level AhR^N^ (Fig. 1o). Notably, the number of effector CD8^+^T cells was the highest in MC38 model, yet the lowest in Ag104Ld model (Fig. 1o). Western blot assays using nuclear, cytoplasmic and membranous proteins showed that AhR expression was high in the nucleus of MC38 but almost undetectable in Ag104Ld cells (Supplementary Fig. 14). PD-L1 was expressed on the membrane of the three cancer cells with the highest level in Ag104Ld cells (Supplementary Fig. 14).

To further dissect the underlying mechanisms, RNA-seq data of 255 lung adenocarcinoma and 251 lung squamous cell carcinoma samples were downloaded from The Cancer Genome Atlas (TCGA) database and were divided into AhR-high and AhR-low groups, using the median expression level as a cutoff value. ESTIMATE (https://bioinformatics.mdanderson.org/estimate/) was applied to assess the infiltration of immune cells in tumor samples. We found that AhR^High^ samples had higher immune scores than AhR^Low^ samples (Supplementary Fig. 15a). The Cancer Immunome Database (TCIA) (https://tcia.at/home) that provides comprehensive immunogenomic analysis results was applied, and we found that AhR^High^ patients were more likely to benefit from inhibitors of CTLA-4, PD-1/PD-L1/PD-L2 (Supplementary Fig. 15b). We previously showed that AhR could bind *PD-L1* promoter in cells exposed to tobacco carcinogen benzo(a)pyrene (BaP).^7^ In the absence of BaP, AhR overexpression increased while AhR suppression inhibited, *PD-L1* promoter-driven luciferase activity (Supplementary Fig. 16a, b). By chromatin immunoprecipitation (ChIP) and real-time reverse transcription-polymerase chain reaction (RT-PCR), we found that AhR directly bound the promoter of *PD-L1* at −700 to −100 bp upstream of its transcription start size (Supplementary Fig. 16c), confirming that AhR is able to control *PD-L1* transcription. Together, the roles of AhR in regulating PD-L1 expression and immune cell infiltration may contribute to its significance in predicting response of NSCLCs to ICIs.

Efforts have been made to identify accurate biomarkers for prognostication of ICIs in treating cancers, but PD-L1,^3,4^ tumor mutation burden (TMB),^8^ or mismatch-repair status^9^ remain unsatisfactory, and the integrative method^10^ needs further investigation. Our findings here indicate that AhR^N^PD-L1^M^ is an accurate, simple, and cheap biomarker for immunotherapy in treating NSCLCs.

## Supplementary information

Supplementary information

## Data Availability

The data that support the findings of this study are available from the corresponding author upon reasonable request.
