# Supplementary material for: Nuclear AhR and membranous PD-L1 in predicting response of non-small cell lung cancer to PD-1 blockade
Source: Signal Transduct Target Ther. 2023 May 29;8:191. doi: 10.1038/s41392-023-01416-5 (PMC10225463; doi:10.1038/s41392-023-01416-5)
Supplement: Supplementary file 1 — Supplementary information [file 41392_2023_1416_MOESM1_ESM.pdf]

Supplementary Information for

Nuclear AhR and membranous PD-L1 in predicting response of non-small cell lung cancer to PD-1 blockade

Si-Chong Han,[1*] Gui-Zhen Wang,[1*] Ya-Ning Yang,[1*] Wen-Feng Fang,[2*] Bei-Bei Sun,[1*] Jian-Dong Zhang,[3] Hua-Qiang Zhou,[2] Li Zhang,[2] Yan Wang,[1] Guang-Biao Zhou[1]

This file contains Methods, 6 supplementary tables and 16 supplementary figures.

Methods

Patients and treatment

The institutional ethical review boards of the three hospitals approved this retrospective study for analyzing anonymous data, and the requirement of informed consent was waived. A total of 168 FFPE samples were collected from patients with histologically or cytologically confirmed advanced NSCLC. The patients were then treated with pembrolizumab at a dose of 200 mg every 3 weeks. These included 118 (70.2%) males, 95 (56.5%) smokers, and 148 (88.1%) with stage IV disease. The baseline demographic characteristics were generally similar between the training and validation groups (Supplementary Tables 1 – 4). Computed tomography (CT) of the chest and abdomen and brain magnetic resonance imaging (MRI) scans were performed every 6 weeks until progressive disease (PD), and the response of complete response (CR),

partial response (PR), stable disease (SD), or PD was evaluated according to the response evaluation criteria in solid tumors (RECIST) version 1.1. The primary endpoint progression-free survival (PFS) was defined as the duration from initiation of therapy to the date of documented disease progression by the investigator or death from any cause. Overall survival (OS) was the period from the date of treatment to death or the last follow-up date.

Multiplex immunohistochemistry and multispectral imaging

The Multiplex immunohistochemistry (mIHC) assays were conducted to detect the cellular localization and expression levels of AhR, ARNT and PD-L1 by using the PANO 4-plex IHC kit (PN130721AD, Panovue, Beijing, China) according to the manufacturer's recommendations, and the investigators were blinded to the response and prognosis of PD-1 antibody-treated patients. Specimens were incubated with anti–PD-L1 (clone 22C3, #M3653, DAKO; #64988, Cell Signaling Technology), anti-AhR (#HPA029723, Sigma; sc-133088, Santa Cruz), anti-ARNT (#5537, Cell Signaling Technology) and anti-CD8 (#98942, Cell Signaling Technology) antibodies, and TSA visualization and signal amplification were performed with Opal TSA Plus (dilution ratio 1:100) (Supplementary Table 5). The multiplex staining process ended with DAPI staining of cell nuclei, and the slides were covered by anti-fluorescence-quenching sealing agent and coverslips.

Slides were scanned and visualized by the PerkinElmer Mantra Quantitative Pathology Imaging System (Waltham, Massachusetts, USA) with the same exposure time at 20× magnification. Five random areas of each sample without hemorrhage, necrosis or detachment were selected. The unmixing of multispectral images and the calculation of staining-positive cell densities, which were expressed as the average number of positive cells per square millimeter, were

performed by Perkin Elmer in Form Image Analysis software (version 2.4.2).

Cutoff value identification and model construction

X-tile (Yale University, version 3.6.1) was used to define the optimal cutoff value of each target marker according to patient PFS. Next, we took advantage of Kaplan–Meier survival analysis and the log-rank test for PFS, identifying 7 variables with statistical significance to construct the prognostic model. Then, 5 powerful predictive markers were determined from the 7 chosen variables by least absolute shrinkage and selection operator (LASSO) logistic regression using the Sangerbox tools (http://vip.sangerbox.com/), of which 3 protective factors were selected.

Cell culture and RNA extraction

The human normal bronchial epithelial cell line 16HBE, lung fibroblast cell line HLF, and the murine lung cancer Lewis lung carcinoma (LLC) line, colon cancer line MC38 and fibrosarcoma Ag104Ld cells were cultured in DMEM supplemented with 10% fetal bovine serum (FBS) and 1% penicillin-streptomycin. The total RNA was isolated using the TRIZOL Reagent (Invitrogen, Frederick, MD, USA) and the phenol-chloroform extraction method according to the manufacturer's instruction. Total RNA was annealed with random primers at 65 ℃ for 5 min. The cDNA was synthesized using a 1st-STRAND cDNA Synthesis Kit (Fermentas, Pittsburgh PA, USA). Quantitative real-time PCR was carried out using SYBR Premix ExTaqTM (Takara Biotechnology, Dalian, China). Chromatin immunoprecipitation (ChIP) assay was performed using AhR-immunoprecipitated DNA samples and primers listed in Supplementary Table 6.

Animals

C57BL/6 mice were injected with 5×10^5 LLC cells via tail vein, three days after xenograft,

the mice were randomized into groups and intravenously injected with vehicle control or 200 μg an anti-PD-1/anti-PD-L1 antibody every three days for 4 times. Three weeks after the last treatment, the mice were anesthetized with the mixture of oxygen/isoflurane inhalation and scanned by microscopic computed tomography (Micro-CT, PerkinElmer, Waltham, MA). Survival of the mice was evaluated from the first day of anti-PD-1 administration to death or became moribund, and the mice were euthanized by cervical dislocation. Tumor tissues were excised, photographed and subjected to Hematoxylin-eosin (HE) and IHC staining. C57BL/6 and B6C3F1 mice were subcutaneously injected with 5×10^5 MC38 and Ag104Ld cells into their right flank, respectively, randomized into groups, and treated intravenously with control or an anti-PD-L1 antibody four times on days 7, 10, 13 and 16. Tumor size was measured every alternate day with electronic caliper and calculated by the formula: volume (mm^3) = ½ $(width)^2 \times$ length. After 22 days of treatment, the mice were sacrificed, and tumor tissues were excised and subjected to HE and IHC staining.

Immunohistochemistry (IHC) analysis

The mouse cancer tissue specimens were deparaffinized through xylene and graded alcohol, and subjected to heat-induced epitope retrieval in citrate buffer solution. Then the slides were blocked with 5% BSA for 30 min, incubated with primary antibodies at 4 °C overnight, and incubated with secondary antibodies at 37 °C for 90 min. Slides was stained with 3, 3'-diaminobenzidine (DAB, Zhongshan Golden Bridge Biotechnology Co., Ltd, Beijing, China), counterstained with hematoxylin and dehydrated.

Nuclear, cytoplasmic and membranous proteins extraction

Extraction was performed using Minute Plasma Membrane Protein Isolation and Cell

Fractionation Kit (#SM-005, Invent, Eden Prairie, MND, USA). Briefly, cells were resuspended in buffer A with protease inhibitor cocktails, transferred to the filter cartridge and centrifuged at 16000 g for 30 sec. The pellet was resuspend by vigorously vortexing for 10 sed and the suspension was centrifuged at 700 g for 1 min, and the pellet contained cell nucleus. Then, the supernatant was transferred to 1.5 ml microcentrifuge tubes and centrifuged at 16000 g for 30 min. The supernatant was the cytosol fraction and the pellet was the total membrane protein fraction, which was resuspended in 200 µl buffer B by vortexing and centrifuged at 7800 g for 5 min. After washed with cold PBS, the supernatant was centrifuged at 16000 g for 30 min, and the pellet was plasma membrane proteins.

Western blot

Cells were lysed in RIPA buffer (50 mM Tris-HCl pH 7.4, 150 mM NaCl, 0.1% SDS, 1% deoxycholate, 1% TritonX-100, 1 mM EDTA, 5 mM NaF, 1 mM sodium vanadate, and protease inhibitors cocktail) for 30 min. Proteins were subjected to 10% SDS-PAGE, electrophoresed and transferred to PVDF membrane. After blocking with 5% milk, the membrane was incubated primary and secondary antibodies, and detected by Luminescent Image Analyzer LSA 4000 (GE, Fairfield, CO, USA).

Luciferase assay

The promoter of PD-L1 containing AhR binding site was amplified and cloned into the modified pGL3-luciferase vector. The cells were transfected with 1 µg of pGL3-luciferase vector and 50 nM of AhR-specific siRNA (siAhR) or 1 µg HA-AhR together with a Renilla plasmid using Lipofectamine 3000 (Invitrogen) according to the manufacturer's instructions. Luciferase activities were measured using the Dual luciferase reporter assay system (Promega,

Madison, WI, USA).

Antibodies and reagents

Antibodies included anti-human PD-L1 (#13684, Cell Signaling Technology, Beverly, MA, USA), anti-human AhR (#83200, Cell Signaling Technology), anti-mouse PD-L1 (#AF1019, R&D, Minneapolis, MN, USA), anti-β-Actin (#A1978, Sigma, St. Louis, MO, USA), anti-mouse AhR (#sc-133088, Santa Cruz, Dallas, Texas, USA), anti-GAPDH (#60004-1-lg, Proteintech, Wuhan, China), anti-Lamin B1 (#12987-1-AP, Proteintech), anti-Na, K-ATPase α1 (#3010, Cell Signaling Technology), anti-Tubulin (#66031-1-Ig, Proteintech), anti-Ki67 (#ab15580, Abcam, Cambridge, MA, USA).

Statistical analysis

The diagnostic efficiency of different predictive markers was quantified by using the area under the receiver operating characteristic (ROC) curve (AUC), and Delong test was used to compare the performance of two ROC curves. Categorical data were analyzed with the Pearson χ^2 test, and continuous data were analyzed with Student's *t* test and the Mann–Whitney *U* test. Survival outcomes were assessed by using the Kaplan–Meier method, log-rank test, and Cox proportional hazards model, which were utilized to perform the univariate and multivariate analyses. Correlations between predictive markers were analyzed by Spearman's rank correlation test. Variables that achieved a level of significance of $P < 0.05$ in the univariable analysis were incorporated into the multivariable model. Analyses were implemented with SPSS software (version 24.0, IBM, Armonk, NY, USA) and GraphPad Prism software (version 5.0, GraphPad Software, San Diego, California, USA). $P < 0.05$ indicated a statistically significant difference.

Supplementary Table 1. The clinicopathological characteristics of the 168 patients.

Characteristics	Total, n (%)	Training cohort, n (%)	Validation cohort 1, n (%)	Validation cohort 2, n (%)	*P* value*
**Total**	168 (100)	65 (100)	64 (100)	39 (100)	
**Gender**					0.0733
Male	118 (70.2)	44 (67.7)	41 (64.1)	33 (84.6)	
Female	50 (29.8)	21 (32.3)	23 (35.9)	6 (15.4)	
**Age**					0.501
≥ 60	95 (56.5)	34 (52.3)	36 (56.3)	25 (64.1)	
< 60	73 (43.5)	31 (47.7)	28 (43.7)	14 (35.9)	
**Smoking status**					0.0744
Smoker	95 (56.5)	32 (49.2)	35 (54.7)	28 (71.8)	
Non-smoker	73 (43.5)	33 (50.8)	29 (45.3)	11 (28.2)	
**Pathological stage**					0.3814
I-II	5 (3.0)	1 (1.5)	1 (1.5)	3 (7.7)	
III	15 (8.9)	6 (9.2)	5 (7.8)	4 (10.2)	
IV	148 (88.1)	58 (89.3)	58 (90.7)	32 (82.1)	
**Histology**					0.0787
Adenocarcinoma	111 (66.1)	44 (67.7)	37 (57.8)	30 (76.9)	
Squamous cell carcinoma	52 (31.0)	17 (26.1)	26 (40.7)	9 (23.1)	
Others	5 (2.9)	4 (6.2)	1 (1.5)	0 (0.0)	

*Two-sided Student's *t*-test for quantitative and chi-square test for qualitative variables.

Supplementary Table 2. The correlation between clinicopathological characteristics and the proportion of AhR^{N}PD-L1^{M}, AhR^{N}ARNT^{N} and PD-L1^{M} in the training cohort.

Variable	Case, N	AhR^{N}PD-L1^{M}			AhR^{N}ARNT^{N}			PD-L1^{M}		
		High	Low	*P*-value*	High	Low	*P*-value*	High	Low	*P* value*
**Gender**										
Male	44	38	6	0.0634	39	5	0.0836	35	9	0.748
Female	21	14	7		15	6		16	5	
**Age**										
≥ 60	34	27	7	0.901	29	5	0.618	23	11	0.592
< 60	31	25	6		25	6		19	12	
**Smoking status**										
Smoker	32	30	2	0.00630	31	1	0.00350	32	0	0.000100
Non-smoker	33	22	11		23	10		10	23	
**Pathological stage**										
I-II	1	1	0	0.857	1	0	0.488	0	1	0.251
III	6	5	1		4	2		5	1	
IV	58	46	12		49	9		37	21	
**Histology**										
Adenocarcinoma	45	34	11	0.347	36	9	0.512	24	21	0.135
Squamous cell carcinoma	16	14	2		14	2		13	3	
Others	4	4	0		4	0		2	2	

*Two-sided Chi-square test was used to compare two groups of categorical variables. *P*<0.05 indicated statistical significance.

Supplementary Table 3. The correlation between clinicopathological characteristics and the proportion of AhR^{N}PD-L1^{M}, AhR^{N}ARNT^{N} and PD-L1^{M} in the validation cohort 1.

Variable	Case,N	AhR^{N}PD-L1^{M}			AhR^{N}ARNT^{N}			PD-L1^{M}		
		High	Low	*P*-value*	High	Low	*P*-value*	High	Low	*P*-value*
**Gender**										
Male	41	24	17	0.0682	19	22	0.111	24	17	0.409
Female	23	8	15		6	17		11	12	
**Age**										
≥ 60	36	20	16	0.314	17	19	0.129	22	14	0.242
< 60	28	12	16		8	20		13	15	
**Smoking status**										
Smoker	35	22	13	0.0238	19	16	0.00610	24	11	0.0142
Non-smoker	29	10	19		6	23		11	18	
**Pathological stage**										
I-II	1	0	1	0.431	0	1	0.683	1	0	0.643
III	6	4	2		2	4		3	3	
IV	57	28	29		23	34		31	26	
**Histology**										
Adenocarcinoma	35	14	21		11	24		18	17	
Squamous cell carcinoma	28	17	11	0.158	14	14	0.234	16	12	0.593
Others	1	1	0		0	1		1	0	

*Two-sided Chi-square test was used to compare two groups of categorical variables. *P*<0.05 indicated statistical significance.

Supplementary Table 4. The correlation between clinicopathological characteristics and the proportion of $AhR^{N}PD\text{-}L1^{M}$, $AhR^{N}ARNT^{N}$ and $PD\text{-}L1^{M}$ in the validation cohort 2.

Variable	Case, N	$AhR^{N}PD\text{-}L1^{M}$			$AhR^{N}ARNT^{N}$			$PD\text{-}L1^{M}$		
		High	Low	*P*-value*	High	Low	*P*-value*	High	Low	*P*-value*
**Gender**										
Male	34	16	18	0.768	23	11	0.576	24	10	0.176
Female	5	2	3		4	1		2	3	
**Age**										
≥ 60	25	13	12	0.328	17	8	0.824	19	6	0.0985
< 60	14	5	9		10	4		7	7	
**Smoking status**										
Smoker	28	14	14	0.442	20	8	0.635	20	8	0.314
Non-smoker	11	4	7		7	4		6	5	
**Pathological stage**										
I-II	3	1	2	0.574	2	1	0.667	3	0	0.119
III	4	1	3		2	2		4	0	
IV	32	16	16		23	9		19	13	
**Histology**										
Adenocarcinoma	30	12	18	0.159	19	11	0.145	19	11	0.420
Squamous cell carcinoma	9	6	3		8	1		7	2	

*Two-sided Chi-square test was used to compare two groups of categorical variables. $P<0.05$ indicated statistical significance.

Supplementary Table 5. Antibodies used in mIHC and Staining Conditions

Markers	Manufacture	Catalog number	Species	Dilution	Opal fluorophores
AhR	Sigma	HPA029723	Rabbit polyclonal	1:1200	Opal520
AhR	Santa Cruz	sc-133088	Mouse monoclonal	1:400	Opal520
ARNT	Cell Signaling Technology	5537	Rabbit monoclonal	1:1000	Opal570
PD-L1	DAKO	M3653	Mouse monoclonal	1:1400	Opal650
PD-L1	Cell Signaling Technology	64988	Mouse monoclonal	1:500	Opal650
CD8α	Cell Signaling Technology	98941	Rabbit monoclonal	1:500	Opal570

Supplementary Table 6. Primers used in the study.

Target	Forward primer (5'→3')	Reverse primer (5'→3')
GAPDH	GAAGGTGAAGGTCGGAGTC	GAAGATGGTGATGGGATTTC
PD-L1	AGTGGTAAGACCACCACCACCAAT	TCATTTGGAGGATGTGCCAGAGGT
**ChIP**		
PD-L1	CTGAAAGCTTCCGCCGATT	CTACCTGCAGGCGGACAGA
**siRNA**	**Sequence 1 (5'→3')**	**Sequence 2 (5'→3')**
siAhR	GGAUUAAAUUAGUUUGUGAdTdT	UCACAAACUAAUUUAAUCCdAdA

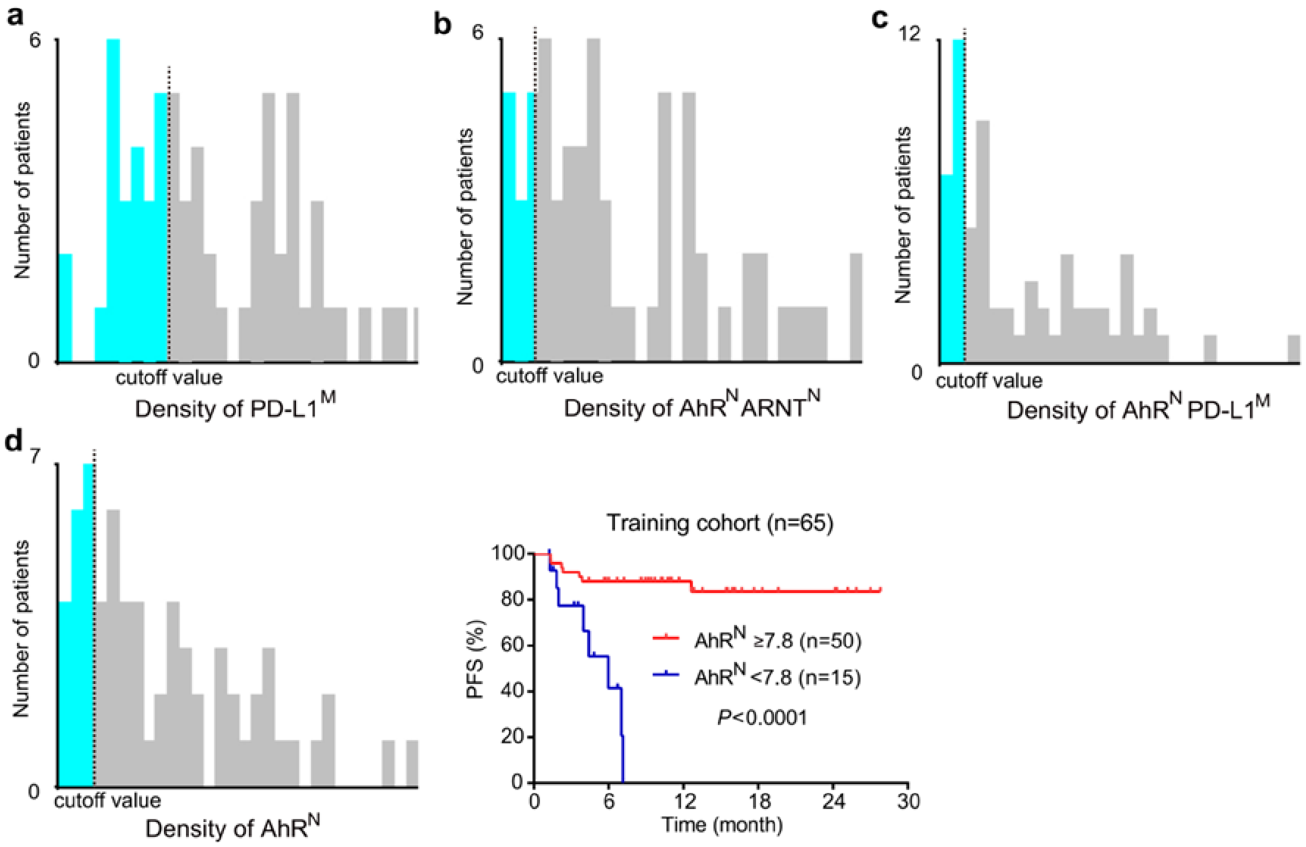

Supplementary Fig. 1. X-tile plots of PD-L1^{M}, AhR^{N}, AhR^{N}ARNT^{N} and AhR^{N}PD-L1^{M} and corresponding Kaplan-Meier survival analysis of AhR^{N} based on patient PFS in the training cohort. Related to Fig. 1b. The optimum density cutoff of PD-L1^{M} (a), AhR^{N}ARNT^{N} (b), AhR^{N}PD-L1^{M} (c), and AhR^{N} and the Kaplan-Meier survival curve of patient PFS corresponding the optimum density cutoff of AhR^{N} (d), are shown. Bar graph shows the cutoff value of each variable. *P* value of Kaplan-Meier survival analysis was calculated by log-rank test.

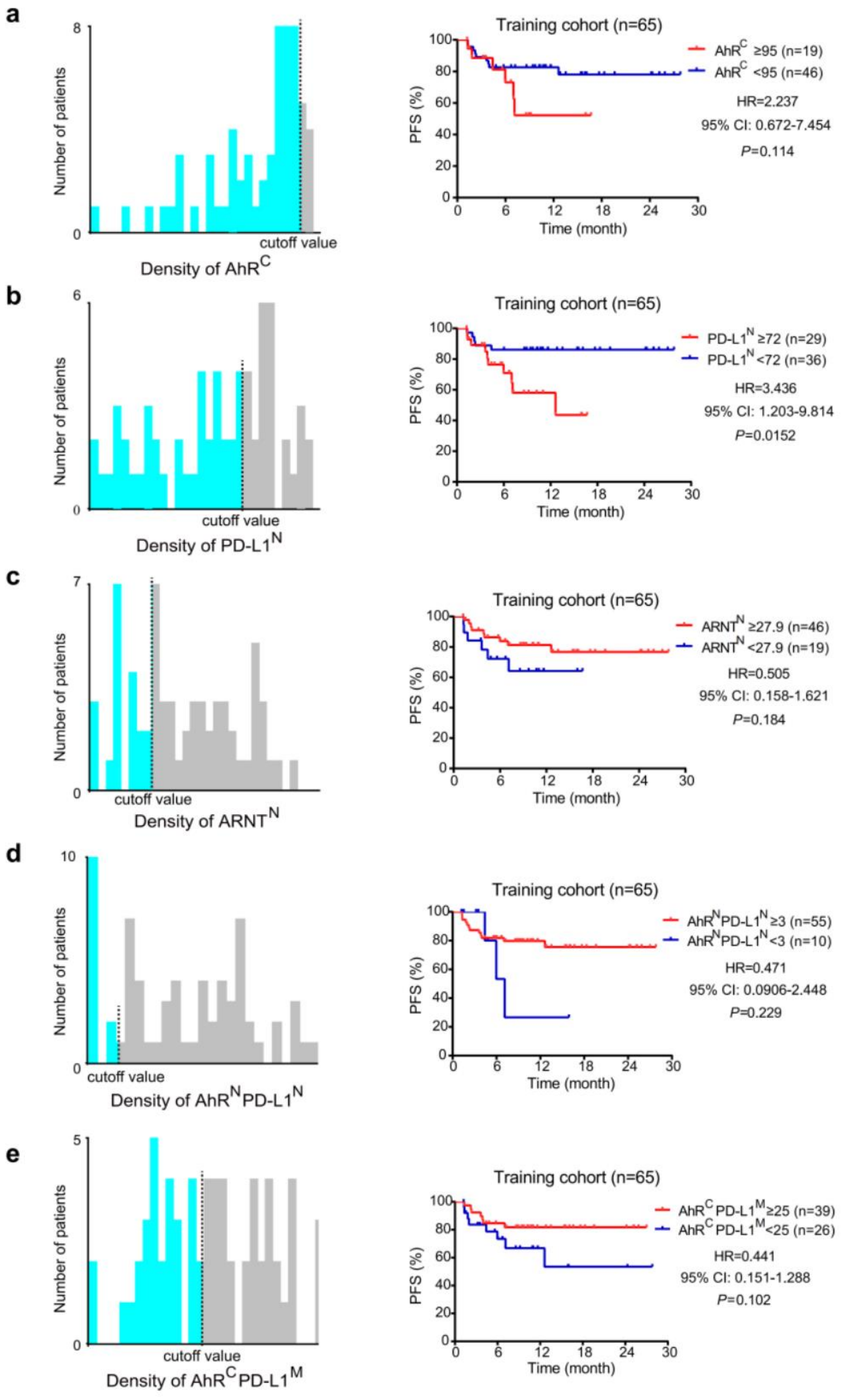

Supplementary Fig. 2. X-tile plots of AhR^{C} (a), PD-L1^{N} (b), ARNT^{N} (c), AhR^{N}PD-L1^{N} (d) and AhR^{C}PD-L1^{M} (e) and corresponding Kaplan-Meier survival analysis based on patient

PFS in the training cohort. Bar graph shows the cutoff value of each variable. *P* value of Kaplan-Meier survival analysis was calculated by log-rank test.

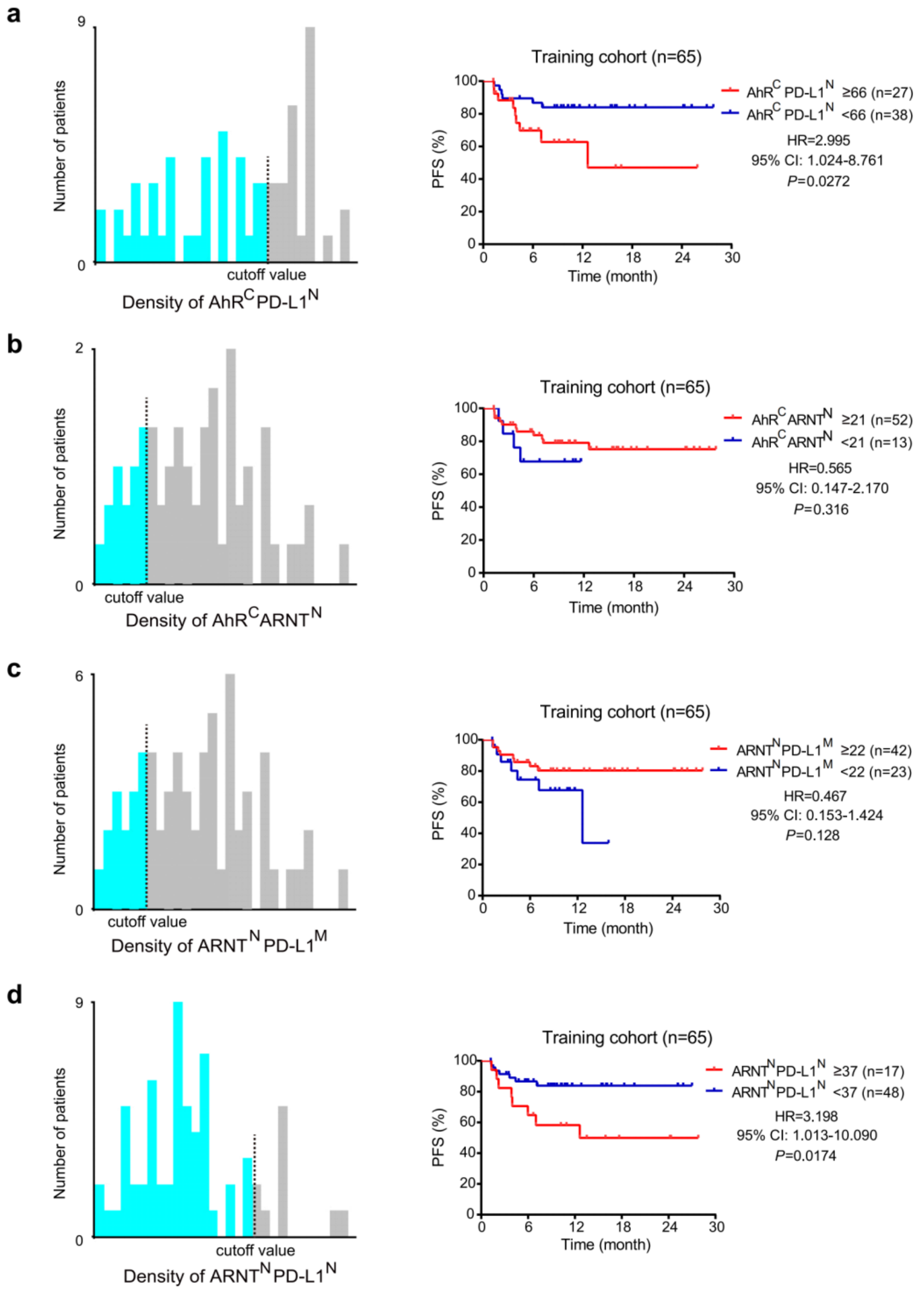

Supplementary Fig. 3. X-tile plots of AhR^{C}PD-L1^{N} (a), AhR^{C}ARNT^{N} (b), ARNT^{N}PD-L1^{M} (c) and ARNT^{N}PD-L1^{N} (d), and corresponding Kaplan-Meier survival analysis based on patient PFS in the training cohort. Bar graph shows the cutoff value of each variable. *P* value of Kaplan-Meier survival analysis was calculated by log-rank test.

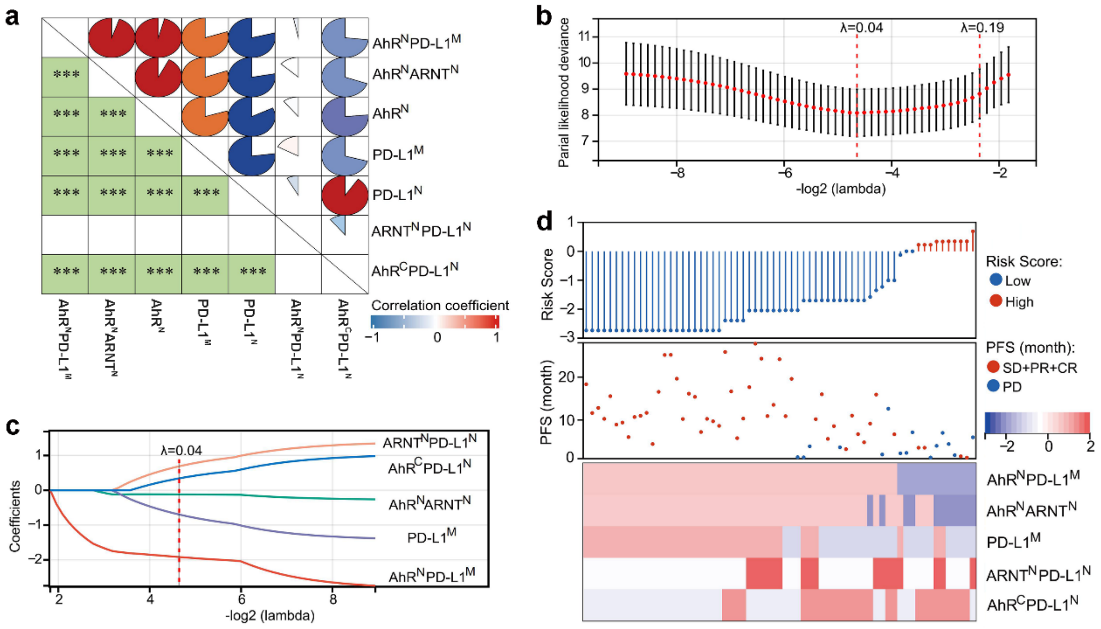

Supplementary Fig. 4. Model construction with the LASSO logistic regression. (a) The correlation between any two variables of AhR^{N}PD-L1^{M}, AhR^{N}ARNT^{N}, AhR^{N}, PD-L1^{M}, PD-L1^{N}, ARNT^{N}PD-L1^{N} and AhR^{C}PD-L1^{N}. (b) Through 5-time cross-validation, λ with the least error was chosen (λ=0.04). (c) Coefficient profiles of the yielded 5 robust immunotherapeutic markers by the LASSO logistic regression. (d) Risk score of 65 NSCLC patients in the training cohort was calculated by the formula derived from the LASSO logistic regression. Heatmaps show the status of each variable multiplied by its coefficient, indicating the correlation between the proportion of AhR^{N}PD-L1^{M}-, AhR^{N}ARNT^{N}-, PD-L1^{M}-, ARNT^{N}PD-L1^{N}- and AhR^{C}PD-L1^{N}-positive cells and patient PFS.

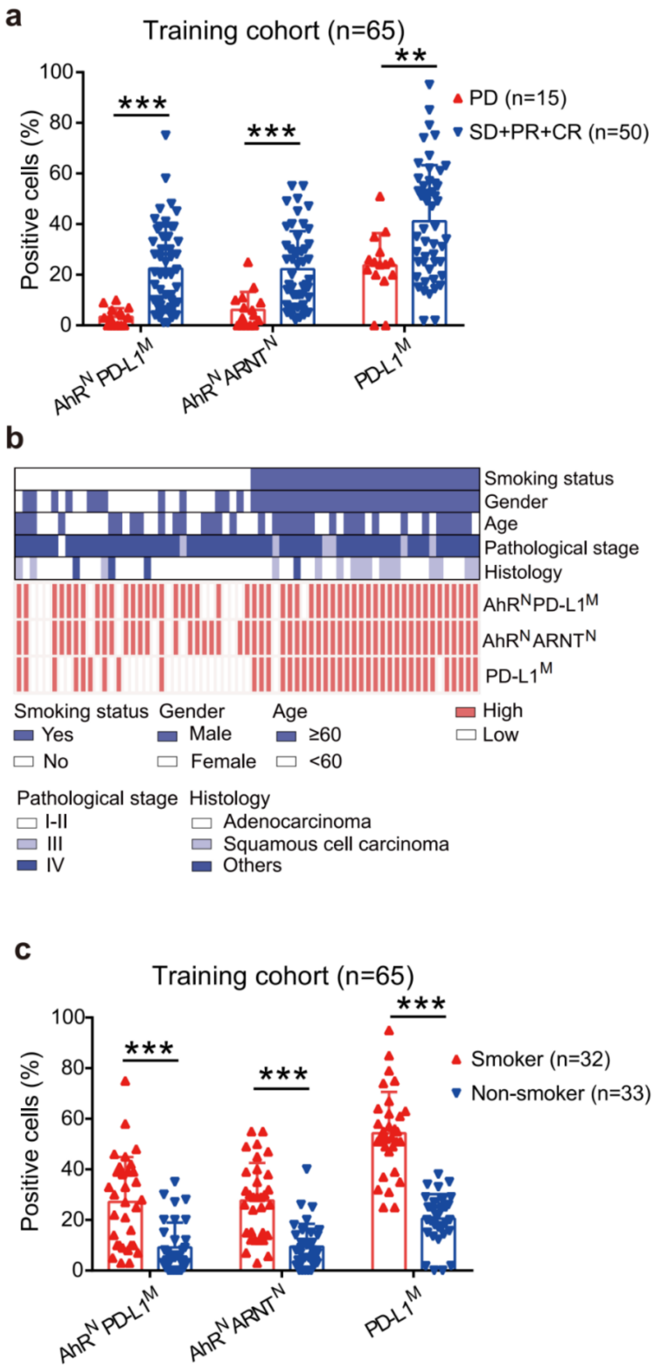

Supplementary Fig. 5. The characteristics of AhR^{N}PD-L1^{M}, AhR^{N}ARNT^{N} and PD-L1^{M} in patients with different clinicopathologic features in the training cohort. (a) The densities of AhR^{N}PD-L1^{M}, AhR^{N}ARNT^{N} and PD-L1^{M} in patients with different level of response to ICI in the training cohort. (b) Clustering of patients in the training cohort based on their clinicopathologic features and the heterogeneous status of AhR^{N}PD-L1^{M}, AhR^{N}ARNT^{N} and

PD-L1^{M}. High, the density of one variable was greater than its cutoff value. Low, the density of one variable was less than its cutoff value. (c) The densities of the 3 variables in patients with different smoking status. The statistical significance was assessed by two-sided Student's *t*-test and *P* value of Kaplan-Meier survival analysis was calculated by log-rank test. ***P* < 0.01 and ****P* < 0.001.

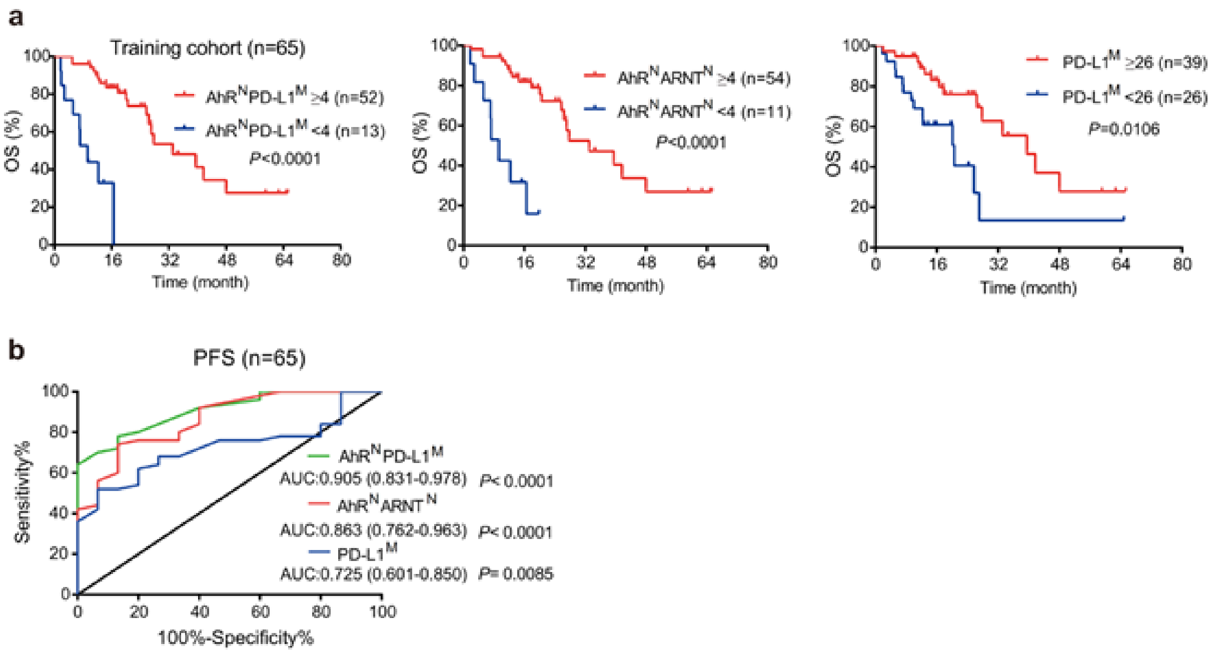

Supplementary Fig. 6. Association between the expression of AhR^{N}PD-L1^{M}, AhR^{N}ARNT^{N} and PD-L1^{M} and overall survival of the 65 NSCLC patients of the training cohort. (a) Kaplan-Meier survival analysis of patient OS according to the status of the 3 variables. (b) ROC curve based on the PFS of patients in the training cohort. *P* value of Kaplan-Meier survival analysis was calculated by log-rank test.

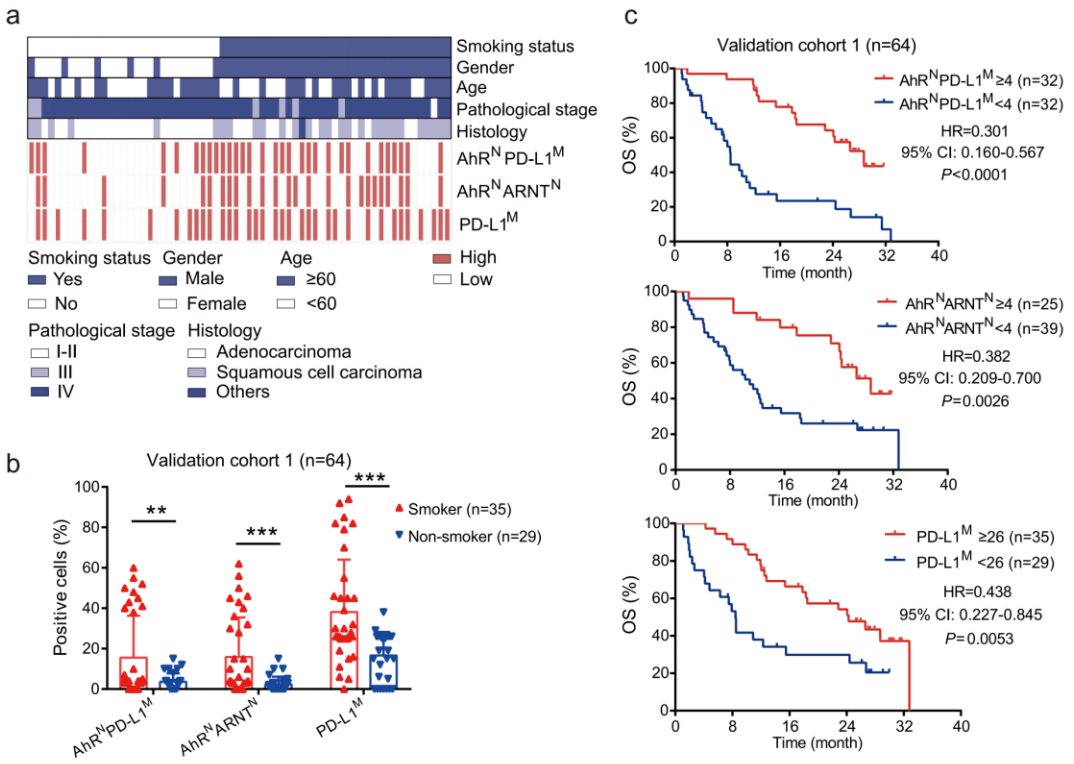

Supplementary Fig. 7. The characteristic of AhR^{N}PD-L1^{M}, AhR^{N}ARNT^{N} and PD-L1^{M} and their performance in predicting patient survival in the validation cohort 1. (a) Clustering of patients in the validation cohort 1 based on their clinicopathologic features and the heterogeneous status of AhR^{N}PD-L1^{M}, AhR^{N}ARNT^{N} and PD-L1^{M}. High, the density of one variable was greater than its cutoff value. Low, the density of one variable was less than its cutoff value. (b) The percentage of cells positive for the 3 variables in patients with different smoking status in the validation cohort 1. (c) Kaplan-Meier analysis of the OS of patients according to the status of AhR^{N}PD-L1^{M}, AhR^{N}ARNT^{N} and PD-L1^{M} in the validation cohot 1. The statistical significance was assessed by two-sided Student's *t*-test. *P* value of Kaplan-Meier survival analysis was calculated by log-rank test. $**P < 0.01$ and $***P < 0.001$.

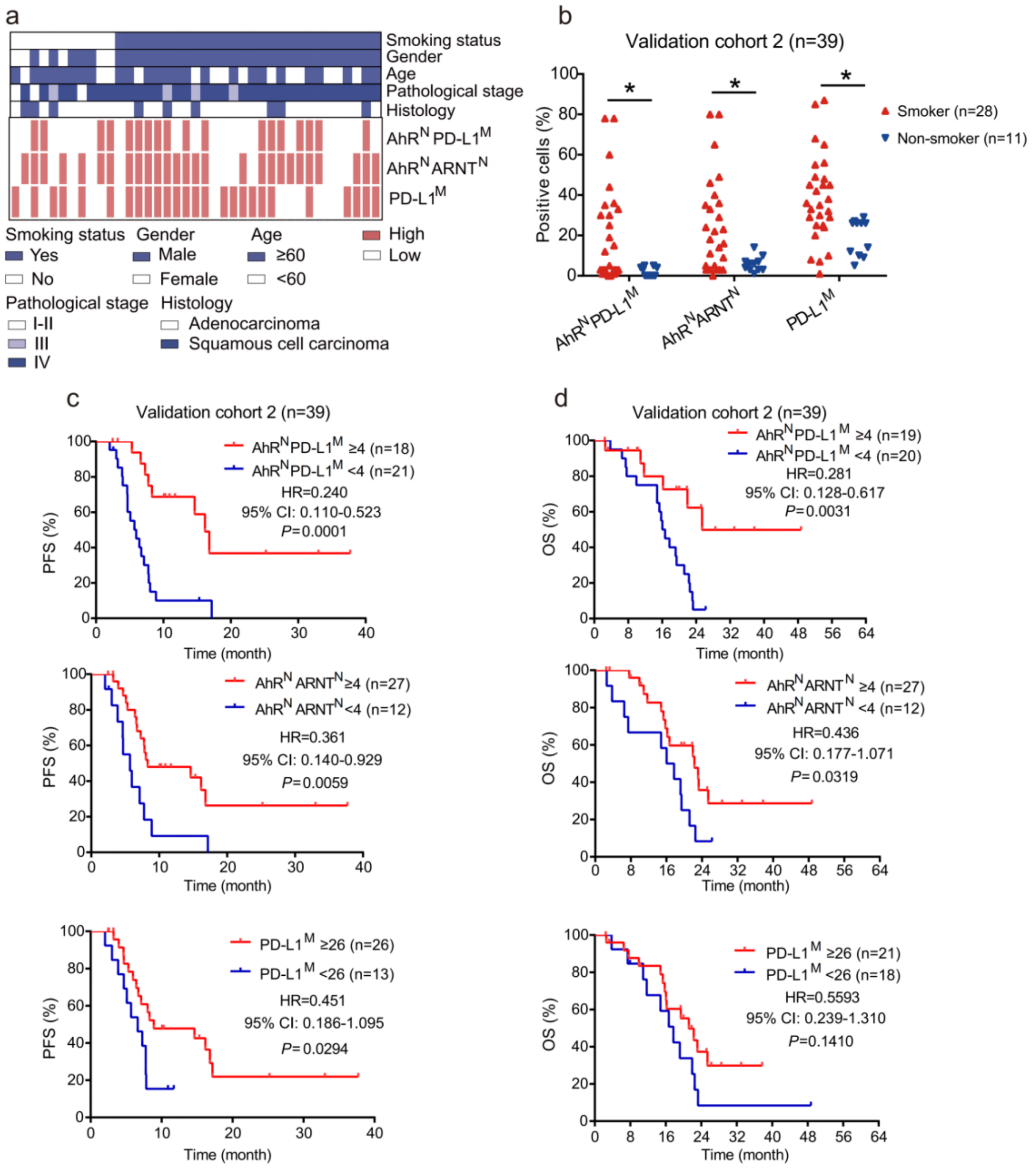

Supplementary Fig. 8. The characteristic of AhR^{N}PD-L1^{M}, AhR^{N}ARNT^{N} and PD-L1^{M} and their performance in predicting patient survival in the validation cohort 2. (a) Clustering of patients in the validation cohort 2 based on their clinicopathologic features and the heterogeneous status of AhR^{N}PD-L1^{M}, AhR^{N}ARNT^{N} and PD-L1^{M}. High, the density of one variable was greater than its cutoff value. Low, the density of one variable was less than its

cutoff value. (b) The percentage of cells positive for the 3 variables in patients with different smoking status in the validation cohort 2. (c) Kaplan-Meier analysis of the PFS of patients in the validation cohort 2. (d) Kaplan-Meier analysis of the OS of patients in the validation cohort 2. The statistical significance was assessed by two-sided Student's *t*-test. *P* value of Kaplan-Meier survival analysis was calculated by log-rank test. *$P<0.05$.

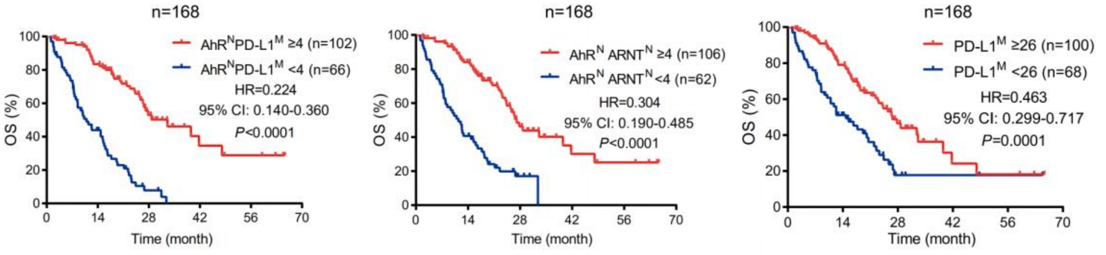

Supplementary Fig. 9. The performance of $AhR^{N}PD\text{-}L1^{M}$, $AhR^{N}ARNT^{N}$ and $PD\text{-}L1^{M}$ in predicting patient OS in all the 168 patients. Kaplan-Meier analysis of the OS of all 168 patients according to the status of $AhR^{N}PD\text{-}L1^{M}$, $AhR^{N}ARNT^{N}$ and $PD\text{-}L1^{M}$. *P* value of Kaplan-Meier survival analysis was calculated by log-rank test.

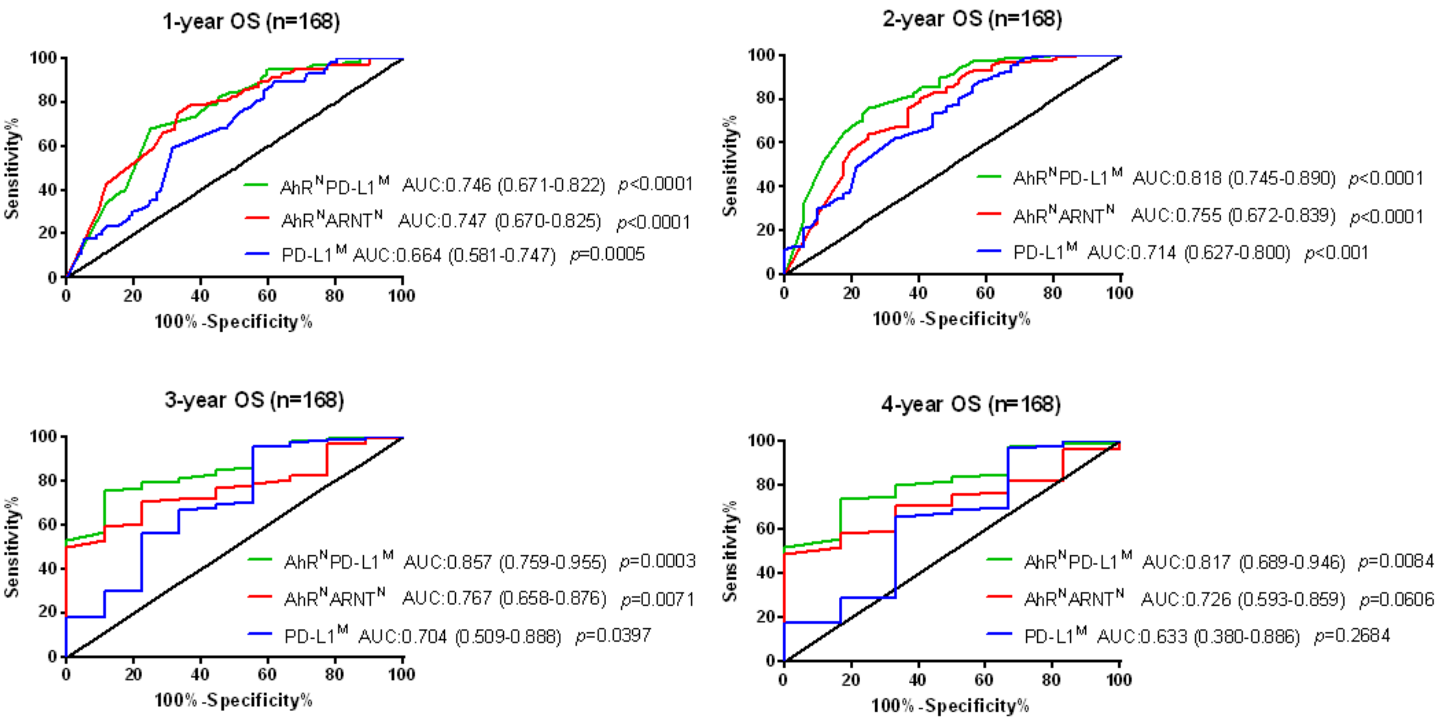

Supplementary Fig. 10. ROC curve based on overall survival (OS) of all 168 patients in 1 to 4 years according to the status of AhR^{N}PD-L1^{M}, AhR^{N}ARNT^{N}, and PD-L1^{M}.

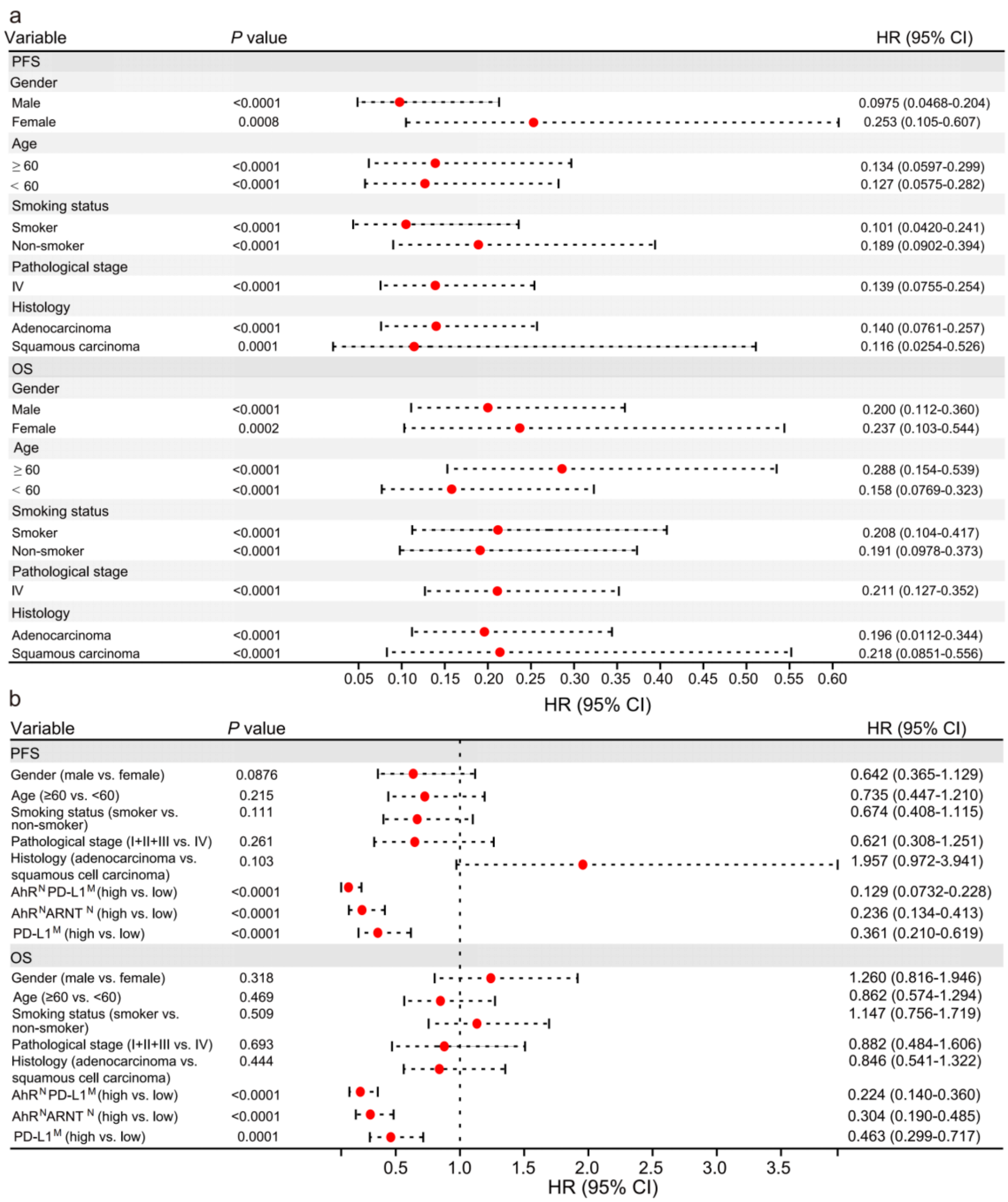

Supplementary Fig. 11. The prognostic effect of AhR^{N}PD-L1^{M} was investigated by Cox regression analyses of the clinicopathological features of NSCLC patients. (a) Forest plots for the stratified COX regression analyses by clinicopathological features of the prognostic effect of AhR^{N}PD-L1^{M}. (b) Univariate Cox regression analyses of AhR^{N}PD-L1^{M}, AhR^{N}ARNT^{N}, PD-L1^{M}, clinicopathological characteristics, and patient survival. The error bars indicate 95% CIs. *P* value of Kaplan-Meier survival analysis was calculated by log-rank test.

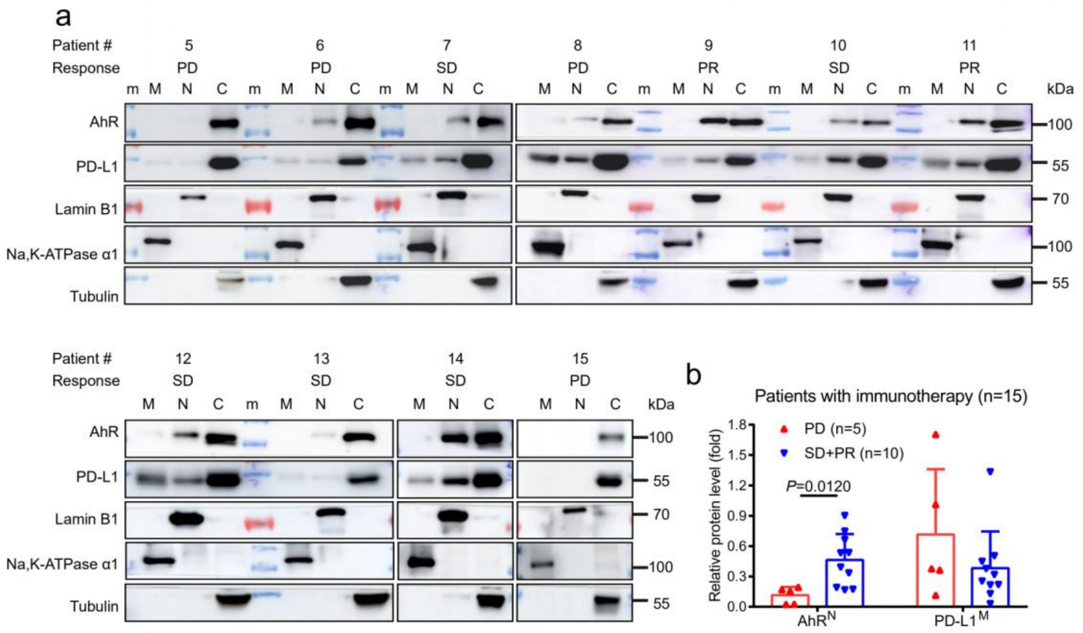

Supplementary Fig. 12. The association between AhR and PD-L1 expression levels in different cellular compartments and the response of NSCLC patients to PD-1 blockade therapy. (a) Western blot analyses of AhR and PD-L1 in nuclear (N), cytoplasmic (C) and membranous (M) compartments using proteins extracted from 15 NSCLC patient specimens. PR, partial response; SD, stable disease; PD, progressive disease; m, marker. (b) The potential association between nuclear AhR (AhR^N) and membranous PD-L1 ($PD\text{-}L1^M$). The relative expression values of AhR^N and $PD\text{-}L1^M$ were the results determined by densitometry analysis normalized to internal controls (Lamin B1 for nuclear compartment and Na, K-ATPase α1 for membranous compartment, respectively). Two-sided Student's *t*-test, **P* = 0.012.

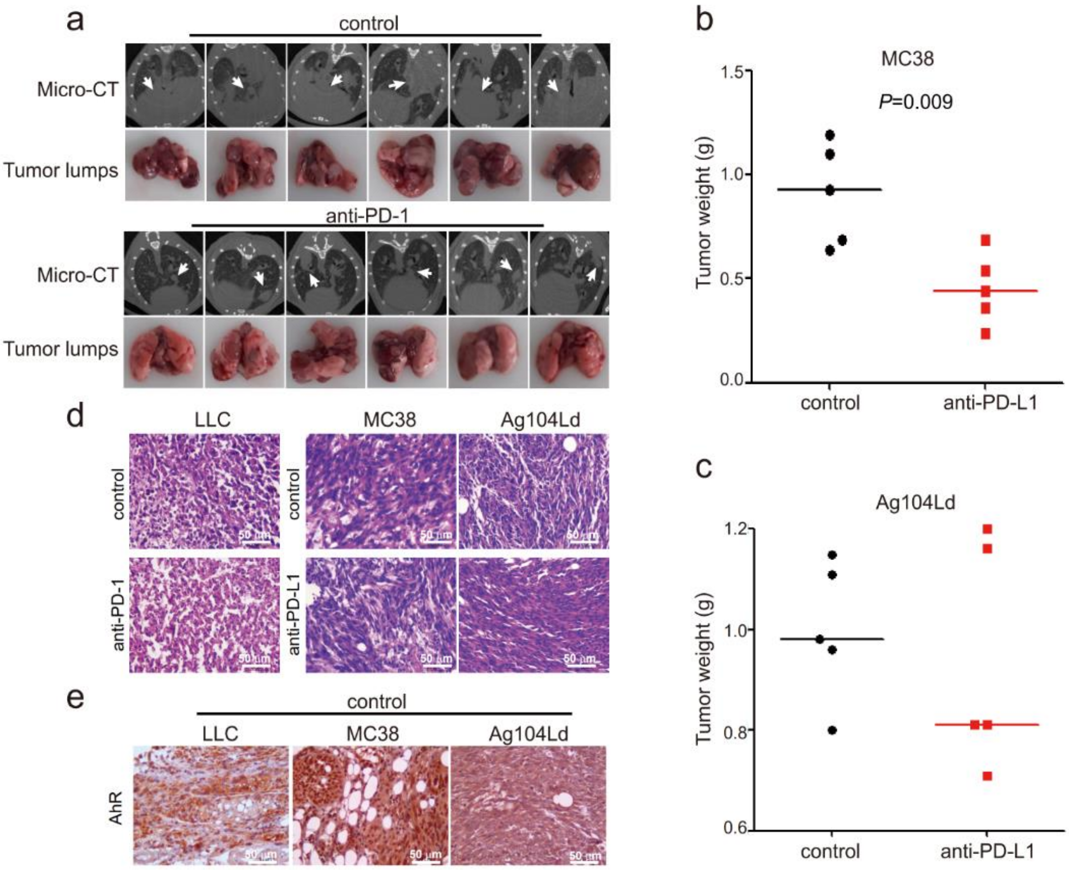

Supplementary Fig. 13. The therapeutic efficacy of immunotherapy on different tumor-bearing mice models. (a) Micro-CT scanning and lung tumor images of LLC-bearing C57BL/6 mice. (b) Tumor weight of tumors isolated from MC38-bearing C57BL/6 mice. n=6 per group. (c) Tumor weight of lumps isolated from Ag104Ld-bearing B6C3F1 mice. n=6 per group. (d) Hematoxylin-eosin (HE) staining of lung sections from three kinds of mouse models. Scale bar = 50 μm. The statistical significance was assessed by two-sided Student's *t*-test.

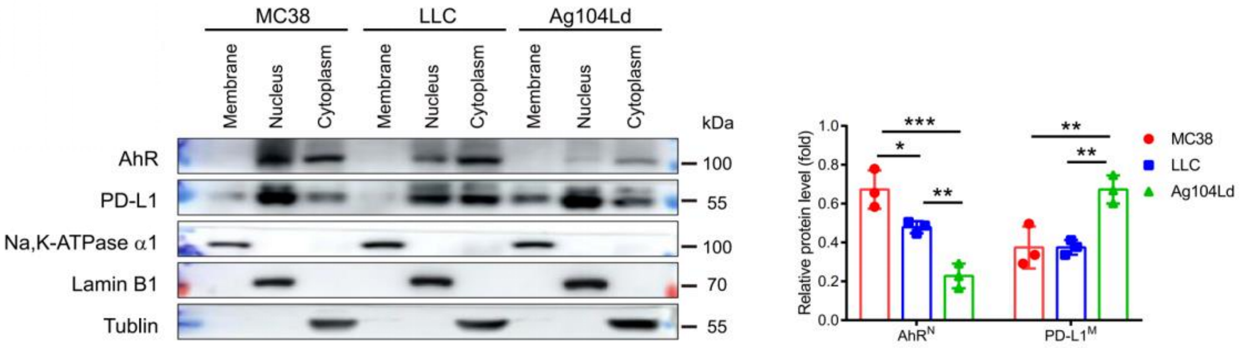

Supplementary Fig. 14. The expression of AhR and PD-L1 in cellular compartments of MC38, LLC and Ag104Ld cells. Nuclear, cytoplasmic and membranous proteins were isolated from the cells and detected by western blot (left panel). The relative expression values of AhR^N and $PD\text{-}L1^M$ were the results determined by densitometry analysis normalized to internal controls (Lamin B1 for nuclear compartment and Na, K-ATPase α1 for membranous compartment, respectively). Two-sided Student's *t*-test, *$P < 0.05$, **$P < 0.01$ and ***$P < 0.001$.

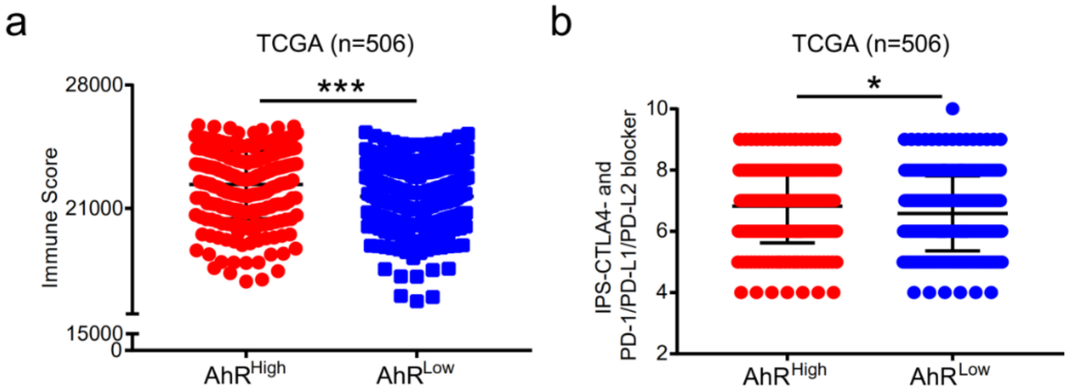

Supplementary Fig. 15. High level of AhR suggests an immunocytic infiltrating phenotype of NSCLCs. (a) ESTIMATE database was used to assess the infiltration level of immune cells in NSCLCs based on the differential expression of AhR. (b) Immunophenoscore (IPS) of NSCLCs with differential expression of AhR predicts response to immunotherapy with

inhibitors of CTLA-4 and PD-1/PD-L1/PD-L2 provided by TCIA database. AhR^{High}, AhR expression is higher than median. AhR^{Low}, AhR expression in this group is lower than median. The statistical significance was assessed by two-sided Student's *t*-test. *P < 0.05 and ***P < 0.001.

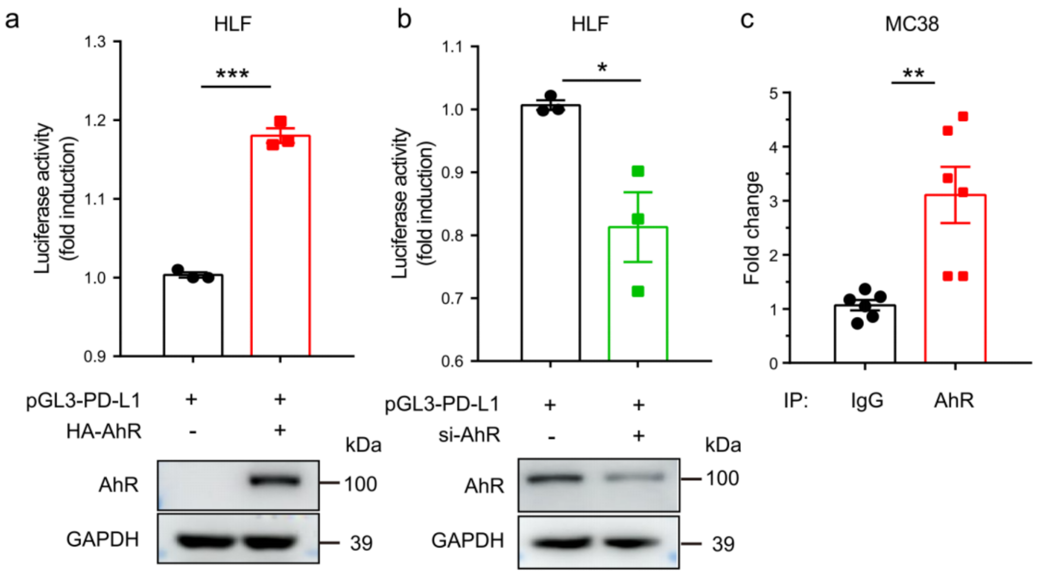

Supplementary Fig. 16. AhR directly binds the promoter of *PD-L1* to promote its transcription. (a) Luciferase assay was performed in HLF cells transfected with *PD-L1* promoter-luciferase reporter construct and HA-*AhR* plasmid. The expression of AhR was detected by western blot. (b) Luciferase assay was performed in HLF cells transfected with *PD-L1* promoter-luciferase reporter construct and siRNA targeting *AhR*. The expression of AhR was detected by western blot. (c) Chromatin immunoprecipitation (ChIP) assay was performed using AhR-precipitated DNA samples of MC38 cells and primers for *PD-L1*. The expression of *PD-L1* was evaluated by real-time RT-PCR. Data are represented as mean ± SD. The

statistical significance was assessed by two-sided Student's *t*-test. *$P < 0.05$, **$P < 0.01$ and ***$P < 0.001$.
